# Machine perfusion for liver transplantation: opportunities and challenges for the next generation of transplant surgeons

**DOI:** 10.3389/frtra.2026.1736191

**Published:** 2026-02-09

**Authors:** Cody L. Mullens, Kumaran Shanmugarajah, Glenn K. Wakam

**Affiliations:** 1 Department of Surgery, University of Michigan, Ann Arbor, MI, United States; 2 Center for Healthcare Outcomes and Policy, University of Michigan, Ann Arbor, MI, United States; 3 UM National Clinician Scholars Program, University of Michigan, Ann Arbor, MI, United States; 4 Department of Surgery, University of Chicago, Chicago, IL, United States; 5 Department of Surgery, Division of Transplant and Hepatobiliary Surgery, University of California San Diego, La Jolla, CA, United States

**Keywords:** hypothermic machine perfusion, liver transplant (LT), machine perfusion, normothermic perfusion, normothermic regional perfusion (NRP)

## Abstract

Machine perfusion is reshaping liver transplantation by turning preservation from passive storage into an active, physiologic intervention. This review synthesizes randomized and real-world evidence on hypothermic oxygenated and normothermic approaches and translates these data into practical guidance for training programs. Across studies, hypothermic oxygenated perfusion reduces ischemic biliary injury, especially in donation after circulatory death, while normothermic perfusion lowers early biochemical injury, decreases early allograft dysfunction, and increases organ utilization through ex situ viability testing. Contemporary cohorts show that “back-to-base” workflows can lessen early complications without increasing ninety-day costs, and that extended preservation, often paired with donor-care unit models, shifts many cases into planned daytime surgery. These operational gains matter for education: daytime cases enable rested faculty, deliberate practice, and graded autonomy. Perfusion broadens the case mix, and the pump itself introduces teachable competencies in setup, troubleshooting, and data-driven accept or decline decisions. Challenges include potential erosion of donor procurement experience, uneven access, and a still-evolving science of viability. We propose a competency-based curriculum spanning setup and safety, physiology, assessment and decision-making, and systems leadership, and outline research priorities as an area of inquiry for future transplantation surgeon-scientists in their training. Aligning training with this platform will improve care and strengthen the transplant workforce.

## Introduction

For more than half a century, static cold storage has been the foundation for allograft storage in liver transplantation (1). This is a relatively simple, low cost, reliable technique, but does have crucial limits on ischemic tolerance and impedes real-time assessment of graft function. These constraints are most challenging for extended criteria donors and donation after circulatory death, where cold ischemia further magnifies hepatocellular and cholangiocyte injury, which are intricately linked to graft dysfunction and ischemic cholangiopathy (1, 2). However, in the last decade, machine perfusion has shifted from theoretical experimental promise to routine clinical practice at many transplant programs (3, 4). This has fundamentally reframed preservation as an active, physiologic intervention that can mitigate graft injury, enable ex situ viability assessment, and reshape timing and logistics of liver transplantation (1, 3, 5).

One overlooked aspect of machine perfusion in liver transplantation, especially as it has become a more routine part of clinical practice, is its impact and implications for transplant surgery trainees (6). In this review, we briefly synthesize the evidence for machine perfusion, discuss how these platforms are pushing transplantation forward, and highlight specific opportunities and challenges these changes create for surgical fellows and junior faculty.

## Biological and logistical rationale

Traditional static cold storage passively slows graft metabolism but cannot clear accumulated metabolites, restore ATP, or provide information that allow surgeons to monitor allograft physiology (1). Hypothermic oxygenated machine perfusion perfuses the liver at low temperature with oxygenated perfusate to replenish energy stores and attenuate reperfusion injury, both of which are of great value especially for donation after circulatory death allografts which are prone to ischemic biliary injury (1, 2). Normothermic machine perfusion operates at near physiologic temperature, facilitates lactate clearance, bile production, and allows evaluation of perfusate hemodynamics and biochemical testing (1, 5, 7, 8). Beyond biology and physiology, both modalities can lengthen safe preservation time, decoupling procurement from graft implantation (4, 9). This has allowed transplant programs to transition from highly time-sensitive operations to allow for more predictable operating room times for the multidisciplinary care that is necessary for liver transplantation (3, 10).

A complementary *in-situ* approach is normothermic regional perfusion (NRP), which restores oxygenated blood flow to abdominal organs after circulatory arrest and before cold preservation. Although mechanistically distinct from ex situ platforms described above, NRP also aims to ameliorate warm-ischemic injury by replenishing energy substrates and clearing metabolites prior to recovery. Its use has expanded significantly in donation after circulatory death and now warrants inclusion in any contemporary preservation discussion alongside hypothermic and normothermic ex situ perfusion (11).

## Current evidence for machine perfusion

Randomized trials have established some distinct strengths for machine perfusion modalities (2, 5, 12). The first multicenter randomized trial comparing normothermic machine perfusion to traditional static cold storage demonstrated lower peak transaminases, reduced early allograft dysfunction, and noninferior graft and patient survival at one year (5). These data also demonstrated that longer preservation times on pump did not adversely impact clinical outcomes for transplant patients. In a separate randomized trial centered around donation after circulatory death using hypothermic oxygenated machine perfusion, data suggested that hypothermic oxygenated machine perfusion significantly reduced biliary complications compared with cold storage of allografts (2). This trial also demonstrated improved early outcomes following liver transplantation. Additionally, the US PROTECT trial compared portable, normothermic perfusion with ischemic cold storage across 300 recipients at 20 programs and demonstrated a lower incidence of early allograft dysfunction, significant reductions in ischemic biliary complications at 6 and 12 months, greater utilization of donation after circulatory death livers, and less histopathologic evidence of ischemia–reperfusion injury, while also meeting its safety noninferiority end point (13).

It is important to note that across randomized trials of normothermic machine transfusion conducted to date, graft and patient survival were largely similar between machine perfusion and standard static cold storage, a finding that may be expected given that these studies largely enrolled standard-risk grafts already deemed suitable for transplantation without ex situ viability testing (4, 12, 13). With growing adoption, particularly for donation after circulatory death, head-to-head comparisons with cold storage are likely to become increasingly limited secondary to loss of clinical equipoise and evidence of utility may be better demonstrated by cohorts transplanting previously declined or higher risk grafts after ex situ viability testing.

More contemporary analyses reinforce and contextualize the signals identified in randomized trials. For example, a recent Cochrane systematic review concluded that cold machine perfusion improves graft survival and reduces serious adverse events and biliary complications, compared to static cold storage (12). Normothermic machine perfusion also consistently demonstrated improved utilization by enabling viability-based organ acceptance of grafts that otherwise would not have been utilized, and had had encouraging effects on early clinical outcomes across different device platforms (14). Other reviews have also emphasized that mechanistic protection of the biliary plexus under hypothermic oxygenated conditions aligns with randomized trial data suggesting reduction in ischemic cholangiopathy, while normothermic platforms also unlock dynamic testing and extend preservation windows (1). These reviews also acknowledge the powerful influence these technologies have on improving transplant center operations, bandwidth, and workflow (3, 4).

NRP is also associated with clinically meaningful improvements in real-world cohorts. A U.S. multicenter donation after circulatory death series reported comparable patient and graft survival but lower rates of ischemic cholangiopathy, fewer biliary complications, reduced early allograft dysfunction, and shorter length of stay for NRP-recovered livers vs. standard super-rapid recovery (15). International registry-scale data similarly demonstrate that abdominal NRP reduces overall biliary complications (including ischemic-type biliary lesions), graft loss, and mortality compared with rapid recovery, reinforcing a mechanistic benefit against warm-ischemia-driven cholangiopathy (11). Similarly, long term transplant outcomes for hypothermic oxygenated machine perfusion are reassuring and support routine adoption into clinical practice (16).

## Real-world practice: utilization, complications, costs, and timing

Outside of trial data, observational evidence has demonstrated how programs are meaningfully integrating perfusion into everyday decision-making and organ acceptance. A multi-center risk-matched cohort analysis of “back-to-base” normothermic machine perfusion was associated with fewer early recipient complications and similar 90-day total costs when compared with standard static cold storage, despite higher acquisition and preservation expenditures (17). This early evidence suggest that improved perioperative trajectories may offset up-front costs of utilizing normothermic machine perfusion. A large European study similarly reported that as normothermic machine perfusion adoption increased, preservation times lengthened with no evidence of adverse outcomes and the proportion of nighttime transplant operations fell dramatically (4). Together, these findings illustrate how viability-based scheduling and extended preservation times can restructure operation timing and workflows for the multidisciplinary staff needed to take care of transplant patients.

These operational observations also align with broader system-level innovations aimed at reducing nocturnal work and unnecessary travel. For example, donor care units (DCU) are dedicated recovery centers focused on efficient and planned procurements which enables a higher proportion of daytime operating room start times, and are associated with more intraoperative extubations and lower transfusion needs all without compromising early outcomes (10). This evidence strengthens the case of machine perfusion's value for patients and transplant teams. For programs weighing the investment, economic models suggest that adopting normothermic machine perfusion to streamline workflow can be cost-effective when disposable costs are moderate and clinical gains reduce complications, length of stay, and resource utilization (14, 17, 18). Importantly, however, the economics of machine perfusion are health-system specific: device and disposable pricing, organ acquisition policies, and reimbursement differ substantially between the United States and Europe, which influences cost-benefit at the case and program level. Published US series and models show clinical gains alongside higher per-case acquisition and disposable costs, with overall 90-day costs ranging from net neutral to increased depending on pricing assumptions and case mix. These realities underscore the need for center-level analyses.

## Aligning transplantation with traditional, higher-performance daytime care

Timing of transplant goes beyond team convenience; it stands to have a measurable impact on organ utilization and team burden (19). For example, liver non-use rates have been documented to be significantly higher at night, especially weekend nights, even after adjusting for donor organ quality (19). Further, there is existing data suggesting that nighttime operations lead to notable surgical team burnout (20). Machine perfusion offers a structural countermeasure by stretching the preservation window and transforming a race against ischemia into a managed interval that can be aligned with daytime operating capacities. The DHOPE-PRO prospective clinical study explicitly evaluated prolonged end-ischemic hypothermic oxygenated machine perfusion to enable next-day daytime liver transplantation (9). These data demonstrated feasibility, excellent one-year survival, and no signal to suggest untoward biliary complications. Together, these data provide proof of concept that preservation can be deliberately extended to achieve more optimal scheduling goals. These scheduling gains are mirrored in real-world series in which most normothermic machine perfusion cases moved into daytime starts, reinforcing machine perfusion as a practical lever for safe, daytime transplantation (21).

Daytime scheduling is likely safer: it reduces *ad hoc* overnight patient drop-offs in the intensive care unit and last-minute acute needs for continuous renal replacement therapy (3, 10). Together, this enables optimized planning and coordination of care and ensures full access to other clinical teams that are needed to take excellent care of liver transplantation patients. Concentrating cases during the daytime supports the development of dedicated liver teams and reduces fatigue, which can lead to better performance (20). As transplant programs continue to iterate on this, the combination of increased perfusion and utilization of DCUs appears to be a pragmatic formula for converting a meaningful share of cases to daytime starts, which tangibly benefits both patients and transplant teams alike.

## Implications for training: opportunities

There is substantial potential upside to machine perfusion itself as a technology and for educational benefits of fellows and newly minted junior faculty (3, 6). First, daytime cases are better teaching cases for trainee learning (10). Rested teams are more readily able to engage in deliberate practice and benefit from graded autonomy (20). In practical terms, rested surgeons training fellows may be more patient and willing to “give away” portions of the operation: recipient hepatectomy, back-table preparation, vascular control and reconstruction, and biliary anastomosis because they are not racing the clock. Second, perfusion expands the case mix trainees see by making higher risk allografts transplantable after ex situ testing, including steatotic grafts and those with longer ischemia time intervals (4, 12). This presents an important opportunity for improving and building trainee judgement across scenarios that otherwise would previously have been rarely considered at many centers. It also allows trainees to link perfusion-phase physiological and biochemical data to postoperative outcomes, turning impressions into repeatable decision frameworks. Third, machine perfusion also opens the door for new roles and career niches in the form of dedicated donor surgery, surgical roles within machine perfusion companies and developing industry relationships to continue pushing the field forward as machine perfusion technology inevitably continues to evolve. Importantly, there is significant learning surrounding placing the organ on the pump, such as cannulation strategy, ensuring secure connections, priming and de-airing, establishing stable flows and pressures, sampling cadence, alarm troubleshooting, and documentation of viability, all constitute a discrete competency that trainees should be taught and assessed on. The practical realities of machine perfusion may attract more trainees to transplantation and increase work satisfaction in a field that has at times struggled with burnout, a maintaining a proper pipeline of trainees, and job placement; particularly as workflows become more predictable and daylight centric (6, 22). Exposure should also include NRP donor recovery and *in-situ* assessment, a discrete competency spanning cannulation strategies, isolation of cerebral circulation, establishment of stable flows and pressures, and serial physiologic monitoring (arterial blood gases, lactate) during perfusion. Without hands-on NRP experience, graduating surgeons will face a steeper learning curve in controlled donation after circulatory death utilization and may be less prepared to lead cross-team choreography with organ procurement organizations and cardiothoracic partners.

## Implications for training: challenges

Despite these real opportunities, there are challenges associated with machine perfusion that training programs must consider and address (6). The rise of local procurement agreements and third-party contractors may reduce travel and improve safety for recipient teams but may shrink fellows' exposure to donor procurement as an unintended consequence (10, 23). This underscores the need for training programs to consider preserving the donor experience through working with organ procurement organizations or potential arrangements with third party contractors (6). Some commentaries have cautioned that the use of local procurement surgeons must not be a synonym for a less qualified surgeon and that deep knowledge of donation after circulatory death physiology, procurement choreography, and communication standards between donor and recipient teams remain crucial to preserve quality (3). In this context, trainees must learn to function and lead within our existing systems even when their own hands or partners are not the ones in the donor operating room. Fellows must be included in decisions about whether to use machine perfusion and whether a graft is viable. Excluding them removes hands-on exposure to a complex, rapidly evolving decision process that they will soon be expected to lead as faculty surgeons.

There are also inherent challenges to teaching trainees about the evolving science of measuring graft function on pump (12). Bile chemistry can be an informative but imperfect predictor of downstream cholangiopathy, and lactate clearance is necessary but not sufficient to understand graft function. Moreover, there are also center-specific experiences with different pump platforms, donor types, and recipient risk which will continue to shape local algorithms until broader, device agnostic consensus standards emerge. Thus, teaching trainees to evaluate and integrate multiple signals to evaluate graft function on pump should be a priority, as should early involvement in ongoing trials and data registry collections that will elevate these decisions from experience and gestalt to reproducible science.

Finally, while trainees are very busy clinically with their skill and clinical development, there are other practical barriers trainees need to consider and understand when it comes to machine perfusion, such as cost and access (17, 18). Real-world cost analyses suggest that acquisition and disposable expenses may be offset by fewer early complications and shorter or more predictable resource use, but those benefits are sensitive to case mix and pricing dynamics that programs do not fully control (17, 18). In parallel, international and regional disparities in adoption reflect not only economics but also regulation, geography, and the distribution of centers with the personnel and infrastructure to operate pumps around the clock. Trainees should be aware that exposure to machine perfusion remains uneven and advocate for visiting rotations or collaborative “pump rounds” that provide cross-institutional experience where needed.

## The need for training curriculum refinement centered around machine perfusion

As the transplant field continues to evolve with machine perfusion so too must the training requirements of our fellows. ASTS should consider incorporating basic knowledge of the various machine perfusion devices, their setups, and how to trouble shoot common problems. The curriculum should be centered around setup and safety, where fellows can learn about cannulation schemas, priming and de-airing, temperature and flow target parameters, alarm logic, and failure modes for at least one of the hypothermic and one of the normothermic platforms. Another aspect of curriculum should be focused on the physiology of machine perfusion, where trainees learn oxygen delivery titration, pharmacotherapy on pump, perfusate composition importance, and protection strategies to preserve endothelium and biliary microcirculation while on pump. Next, trainees need start acquiring experience with assessment and decision-making. While there is no consensus on viability criteria on the pump trainees would benefit from learning to serially assess and synthesize trends across metrics like lactate, bile production, transaminases, perfusate acid-base status, and pump flows and pressures (24). This will provide a much-needed foundation for decisions on using this new technology.

## Robust opportunities for scholarship

The next phase of perfusion science and systems design is especially conducive to trainee leadership and will be able to be translated into meaningful scholarly endeavors for aspiring surgeon-scientists and future academic surgery leaders. Comparative effectiveness studies can define when to choose hypothermic oxygenated machine perfusion vs. normothermic machine perfusion and when to combine them, including sequences that interact with *in situ* normothermic regional perfusion where ethically and operationally appropriate. High-fidelity registries and quality improvement collaboratives that capture standardized perfusion phase signals alongside detailed donor, recipient, and outcome data will enable predictive modeling of viability thresholds that generalize across devices and centers, and multicenter collaborations are increasingly feasible as more programs adopt back-to-base workflows. In the translational science realm, proof-of-concept reports have demonstrated three-day normothermic preservation of a human liver prior to successful transplantation, opening a therapeutic window for ex situ intervention that could transform perioperative pathways if device simplification and automation keep pace (25). On the operations side, health services research can quantify how daytime transplantation affects duty hours, burnout, nursing continuity, and resource use. Other work can also evaluate how local procurement redistributes risk across donor and recipient teams, how reimbursement models can be aligned with real-world cost curves for disposables and length of stay without creating inequities in access, and can evaluate care quality. Perhaps most importantly, the benefits and possible negative impact of machine perfusion on fellowship training have not been quantified and published which creates an extremely important gap in academic knowledge ripe for investigation.

## Conclusion

In summary, machine perfusion has created a substantial paradigm shift in transplantation with the support of robust data. This evolution presents both important opportunities to capitalize on and potential challenges that should be accounted for by training programs. For trainees, this evolution creates richer decision-making opportunities for learning and a more teachable operating environment. It also lends itself to new career niches in donor surgery, perfusion leadership, and data-driven viability science. If programs meet these challenges with thoughtful curricula and rigorous evaluation, machine perfusion will not only modernize organ preservation but also help sustain and grow a workforce capable of delivering on the promise of liver transplantation in the decades ahead.
